# The gut microbiome, immune modulation, and cognitive decline: insights on the gut-brain axis

**DOI:** 10.3389/fimmu.2025.1529958

**Published:** 2025-01-22

**Authors:** Ruyi Zhang, Ning Ding, Xicui Feng, Wenli Liao

**Affiliations:** ^1^ School of Pharmacy, Hubei University of Chinese Medicine, Wuhan, China; ^2^ Basic Medical School, Hubei University of Science and Technology, Xianning, China

**Keywords:** gut microbiota, immune modulation, cognitive dysfunction, gut-brain axis, short-chain fatty acids

## Abstract

The gut microbiome has emerged as a pivotal area of research due to its significant influence on the immune system and cognitive functions. Cognitive disorders, including dementia and Parkinson’s disease, represent substantial global health challenges. This review explores the relationship between gut microbiota, immune modulation, and cognitive decline, with a particular focus on the gut-brain axis. Research indicates that gut bacteria produce metabolites, including short-chain fatty acids (SCFAs), which affect mucosal immunity, antigen presentation, and immune responses, thereby influencing cognitive functions. A noteworthy correlation has been identified between imbalances in the gut microbiome and cognitive impairments, suggesting novel pathways for the treatment of cognitive disorders. Additionally, factors such as diet, environment, and pharmaceuticals play a role in shaping the composition of the gut microbiome, subsequently impacting both immune and cognitive health. This article aims to clarify the complex interactions among gut microbiota, immune regulation, and cognitive disorders, evaluating their potential as therapeutic targets. The goal is to promote microbiome-based treatments and lay the groundwork for future research in this field.

## 1 Introduction

Cognitive dysfunction, characterized by a notable deterioration or impairment in cognitive abilities, has emerged as a critical global health concern, impacting millions of individuals worldwide (1, 2). The prevalence of cognitive dysfunction is on the rise, primarily attributed to the rapid aging of populations (3). Research indicates that approximately 15.5% of individuals aged 60 and older experience cognitive dysfunction, a figure that escalates to 33.1% among those aged 90 and above (4). Furthermore, a study focusing on individuals aged 60 and older in China revealed an overall prevalence rate of 33.59% (5). The financial burden associated with the treatment of Alzheimer’s patients is substantial, with social and economic costs exceeding one trillion yuan (6, 7). These statistics underscore the profound societal and economic ramifications of cognitive impairment, highlighting the urgent need for further research in this critical area.

The human gut microbiota constitutes a complex microbial ecosystem that plays a crucial role in the immune system regulation (8). Research has established a significant connection between gut microbiota and immune function, encompassing the modulation of mucosal immunity, effects on antigen presentation, and the production of metabolites that influence immune responses, all of which can ultimately impact cognitive function (9–11). Furthermore, the interaction between the microbiome and the host’s immune cells can either promote tolerance or provoke inflammation (12). This underscores the dual role of the microbiome in maintaining immune balance while also having the potential to contribute to disease conditions (13–17).

The gut-brain axis encompasses a variety of signaling pathways that interconnected the neural, endocrine, and immune systems, facilitating the bidirectional communication between the gastrointestinal system and the central nervous system (18–20). Increasing evidence suggests that the gut microbiome significantly influences brain function, behavior, and potentially cognitive processes (21–23). Short-chain fatty acids (SCFAs) and other microbial metabolites can traverse the blood-brain barrier, thereby modifying neurotransmission and affecting neuronal activity (24–26). Dysbiosis, characterized by imbalances in gut microbiota, is strongly associated with cognitive decline (27–29). This body of evidence indicates that the gut-brain axis may present promising opportunities for therapeutic interventions.

This review utilized keywords such as “gut microbiome,” “immune regulation,” and “cognitive dysfunction” to synthesize and analyze contemporary literature from databases, including PubMed, Google Scholar, Web of Science, and CNKI. The primary focus was on the impact of dietary composition, environmental influences, and pharmacological treatments on mucosal immunity, systemic immune responses, and inflammatory diseases. Although current research indicates a connection between gut microbiota and cognitive function, our understanding of the mechanisms underlying these interactions remains limited. Additionally, the role of gut microbiota in preventing or mitigating cognitive impairments presents a significant challenge. Consequently, this review aims to bridge this knowledge gap by providing a comprehensive analysis of the relationships among gut microbiota, immune regulation, and cognitive dysfunction. It evaluates the therapeutic potential of targeting gut microbiota as a strategy to combat cognitive decline. The objective is to establish a scientific foundation for innovative treatment approaches that leverage gut microbiota to enhance cognitive health and slow the progression of neurodegenerative diseases. This investigation seeks to elucidate the complex mechanisms through which gut microbiota influences cognitive function and to pave the way for future pioneering research that could lead to effective prevention and treatment of cognitive impairments, ultimately offering hope to millions affected by these conditions.

## 2 Gut microbiome-immune system crosstalk in health and disease

### 2.1 Gut microbiota influence on mucosal immune responses

The gut microbiome encompasses the extensive and intricate assemblage of microorganism residing in the gastrointestinal tract of animals, significantly influencing mucosal immunity through sophisticated interactions with immune cells situated in the lower mucosa of the gut (30–32), as shown in **Table 1**. Recent research highlights the significant influence of gut microbiota on mucosal immunity, demonstrating that specific types of microbiotas can enhance both the development and functionality of immune cells (33). Amoroso et al. have shown that gut microbiota bioregulators can substantially impact mucosal immunity and intestinal inflammation (34). This influence is mediated through mechanisms that interact with host cells, thereby facilitating the maturation and activation of mucosal immune cells. Additionally, Lehmann et al. provide further insights, indicating that gut flora modulates the antigen presentation process by influencing innate lymphocytes (ILC3s), thereby establishing a strong connection with the host’s immune system (35).

**Table 1:** Main classification and function of human gut microbiota.

Types ofmicroorganisms	Key members	Main functions
Bacteria	*Firmicutes*, *Bacteroidetes*, *Actinobacteria*, *Proteobacteria*, *Verrucomicrobia*	Produce short-chain fatty acids, regulate the immune system, inhibit pathogen growth; Synthesize vitamins, maintain intestinal barrier function, etc.
Fungi	Candida albicans, Aspergillus	Participate in the fermentation of carbohydrates; Competing with bacteria for niche; Modulating immune response
Virus	Bacteriophages, Eukaryotic viruses	Regulates bacterial population dynamics, influences the evolution of bacterial genomes, and interacts with the host immune system
Protist	Amoebae, Flagellates	Involved in the ecological balance of the gut, interacting with bacteria and the host immune system
Archaea	*Methanobrevibacter*	It is involved in methane production and affects the intestinal fermentation process

In the field of organ transplantation, Dery et al. have provided valuable insights into the influence of gut microbiota on the immune system (36). Their research underscores the critical role that gut microbes play in modulating immune responses among transplant recipients. Furthermore, attention has turned towards probiotics, with Dowdell et al. demonstrating that Enterococcus and Lactococcus strains, sourced from Thai fermented sausages, exhibit significant probiotic activity (37). These strains have shown protective effects against Clostridium difficile infections, indicating that specific beneficial microbiota can substantially enhance host immune responses.

The therapeutic potential of gut microbiota in the treatment of colitis has attracted significant interest. Chiang et al. investigated the modulation of gut microbiota as a strategy for managing colitis, emphasizing fibroblast growth factor 19 (FGF19) as a promising therapeutic agent (38). In the context of cancer immunotherapy, research by Halsey et al. and Inamura underscores the vital role of gut microbiota in mediating therapeutic responses (39, 40). Their findings indicate that monitoring gut microbiota could enhance the effectiveness of immune checkpoint inhibitors.

### 2.2 Study on the interaction between intestinal flora and immune system

Complex interactions exist between the gut microbiome and the body’s immune and inflammatory responses (41, 42). Recent studies indicate that intestinal microorganisms are crucial for maintaining mucosal barrier function, regulating metabolic processes, and preserving immune balance (43–45). They also play a significant role in the onset, progression, and management of inflammatory bowel disease (IBD) (46). Dysbiosis, characterized by a decrease in beneficial bacteria and an increase in harmful strains, is strongly correlated with the development of IBD (47). For instance, patients with IBD demonstrate a reduction in butyrate-producing bacteria, which disrupts intestinal equilibrium (48). Furthermore, metabolites produced by gut microbiota, such as short-chain fatty acids (SCFAs), enhance gut barrier integrity by activating G protein-coupled receptors and promoting the generation and differentiation of regulatory T cells (TREGs), thereby supporting intestinal homeostasis (49–51). T cell subsets encompass helper T cells (CD4+ T cells) and cytotoxic T cells (CD8+ T cells), both of which play a pivotal role in cognitive function by modulating the blood-brain barrier and neuroinflammatory processes (52, 53). Furthermore, the immune cell repertoire also comprises B cell subsets and natural killer cells (NK cells), whose activities are intricately linked to memory functions (54).

The gut microbiota plays a crucial role in modulating inflammatory responses by influencing the activity of innate lymphocytes (ILCs) and T cells (41). For instance, Lactobacillus metabolizes tryptophan from the intestinal flora into indole-3 acetaldehyde, a metabolite that activates the aromatic hydrocarbon receptor (AhR) (55). This activation leads to the induction of IL-22 expression, which in turn stimulates epithelial cells to produce antimicrobial peptides, thereby supporting mucosal homeostasis. Furthermore, alterations in gut microbiota composition are associated with an increased susceptibility to inflammatory bowel disease (IBD) (56). Notably, certain bacteria, such as *adhesive invasive Escherichia coli* (AIEC), are prevalent in IBD patients, where they adhere to and invade intestinal epithelial cells, promoting the expression of inflammatory factors and contributing to the pathogenesis of IBD (57).

The gut microbiota played a crucial role in influencing immune and inflammatory responses by a variety of mechanisms. These mechanisms include the regulation of immune cell function, the preservation of intestinal barrier integrity, and the production of metabolites that modulate immune responses. These findings open new avenues for innovative therapeutic strategies aimed at the prevention, diagnosis, treatment, and prognosis of disorders related to the immune system.

## 3 Influence of diet on gut microbiota and immunomodulation

Diet significantly influences the gut microbiota, which in turn profoundly affects the body’s immune status (58). Experimental data indicate that a high-fiber diet promotes the growth of beneficial bacteria, particularly short-chain fatty acid-producing bacteria (59–61). The metabolites of these bacteria can modulate immune cell function and elicit anti-inflammatory effects through the activation of G protein-coupled receptors, such as GPR43. For instance, Holscher posits that augmenting dietary fiber consumption can markedly enhance the proliferation of beneficial gut microbiota while concurrently reducing inflammatory marker levels (62). Conversely, a high-fat, high-sugar Western diet can disrupt the gut microbiota, leading to diminished microbial diversity and facilitating the colonization of pathogens, which may result in inflammation and an increased risk of autoimmune diseases (63). Recent studies have shown that participants adhering to a low-fiber, high-fat diet experienced a significant reduction in gut microbiota diversity and an increase in bacterial species associated with inflammatory bowel disease (64).

Short-chain fatty acids (SCFAs), especially butyric acid, have been demonstrated to activate immune cells through the GPR109A receptor, promoting the production of anti-inflammatory cytokines and thereby aiding in the maintenance of intestinal immune tolerance (65). Additionally, dietary fiber intake is closely associated with the composition of the gut microbiota; a high-fiber diet enhances the α-diversity of the SCFA-producing microbiome within the gut (66). Beyond their local effects, SCFAs also modulate the immune response in distal organs via the circulatory system.

The gut microbiota is notably influenced by a high-fat diet (HFD), which also affects immune function (67–69). Research has shown that HFD induces ferroptosis in intestinal regulatory T cells (Treg), a critical initial event leading to the disruption of immune tolerance and the onset of colitis. The ferroptosis of Treg cells results in a reduction of their immunosuppressive capabilities, thereby exacerbating the inflammatory response (70). Furthermore, HFD-induced dysbiosis of the intestinal microbiota increases intestinal barrier permeability, which promotes further activation of resident immune cells and elevates the production of reactive oxygen species (ROS) due to lipid peroxidation and Treg cell death (71). Importantly, the ferroptosis of Treg cells can be counteracted by antioxidant supplementation, such as α-tocopherol, which significantly alleviates colitis symptoms associated with a high-fat diet. These findings reveal novel mechanisms for modulating gut microbiota and immune responses through dietary interventions, suggesting potential therapeutic strategies for the prevention and management of diet-related chronic inflammatory diseases, as shown in **Table 2**. For example, a randomized controlled trial demonstrated that antioxidant vitamin E supplementation improved clinical symptoms while significantly reducing bowel inflammation in patients with colitis (72).

**Table 2:** Effects of different dietary patterns on gut microbiota.

Dietary patterns	Main Features	Effects on the gut microbiome
High-fiber diet	Rich in fruits, vegetables, whole grains and legumes and low in fat and sugar	Increase intestinal microbial diversity, promote the growth of beneficial bacteria, improve the production of short-chain fatty acids (SCFAs), enhance immune function and intestinal barrier function
High-fat diet	High in saturated and trans fats	Reducing gut microbial diversity, increasing bacteria associated with inflammation, decreasing the proportion of beneficial bacteria, increasing intestinal permeability, leading to inflammation and metabolic syndrome
High-sugar diet	Rich in added sugars and refined carbohydrates	Reducing beneficial bacteria and increasing bacteria associated with metabolic diseases may lead to dysregulation of gut microbiota and inflammation, increasing the risk of obesity and diabetes
Mediterranean diet	Rich in fruits, vegetables, whole grains, nuts, olive oil, and fish	Increase gut microbial diversity, promote the growth of beneficial bacteria, improve the production of short-chain fatty acids, reduce inflammation and cardiovascular disease risk
Vegetarian/vegan diet	Free of meat and animal products and rich in fruits, vegetables, legumes, nuts and whole grains	Increases gut microbial diversity, promotes bacteria associated with a plant-based diet, improves short-chain fatty acid production, and reduces inflammation and chronic disease risk
Low carb/ketogenic diet	High in fat, moderate in protein and very low in carbohydrates	Changing gut microbiota composition and increasing bacteria associated with fat metabolism may reduce the proportion of beneficial bacteria and may increase inflammation and intestinal permeability
Fermented food diet	Rich in fermented foods such as yogurt, kimchi, miso and sauerkraut	Increasing the intake of beneficial bacteria and enhancing intestinal barrier function may improve immune function and digestive health
Western diet	High in fat, sugar and salt	Reducing gut microbial diversity, increasing bacteria associated with inflammatory and metabolic diseases, lowering the proportion of beneficial bacteria and increasing the risk of obesity, diabetes and cardiovascular disease

## 4 Exploring the correlation between gut microbes and cognitive dysfunction

The dietary modulation of the gut microbiome not only enhances immune functionality but also influences cognitive processes via the “gut-brain axis,” thereby facilitating a holistic regulation encompassing the gut microbiome, immune response, and cognitive function. The close association between gut microbes and cognitive function has emerged as a significant area of research in Alzheimer’s disease (73). Recent studies indicate that gut microbes influence host cognitive function through a gut-brain axis, which encompasses multiple complex mechanisms, including the production of neuroactive metabolites, modulation of immune responses, and maintenance of gut barrier function (74–76). In the pathophysiology of Alzheimer’s disease, alterations in the abundance of specific intestinal flora are closely linked to disease progression. For instance, amyloid-positive patients with Alzheimer’s disease demonstrate a lower relative abundance of *Proctococcus* and *Bacillus subtilis*, coupled with a higher abundance of *E. coli/Shigella* (77). The results indicate that alterations in gut microbiota and their equilibrium may be crucial in the development of Alzheimer’s disease. The imbalance of gut microbiota can result in elevated concentrations of pro-inflammatory cytokines (including IL-1β and TNF-α), which are conveyed to the central nervous system via the “gut-brain axis,” instigating neuroinflammatory responses. Lipopolysaccharides (LPS) generated by intestinal microbiota have the capacity to traverse the blood-brain barrier through systemic circulation, subsequently activating microglial cells, which can culminate in neuronal injury and a deterioration of cognitive abilities (78–80). Additionally, the gut microbiota may influence normal brain functioning by modulating nutrient absorption and metabolism (81).

**Figure 1:**
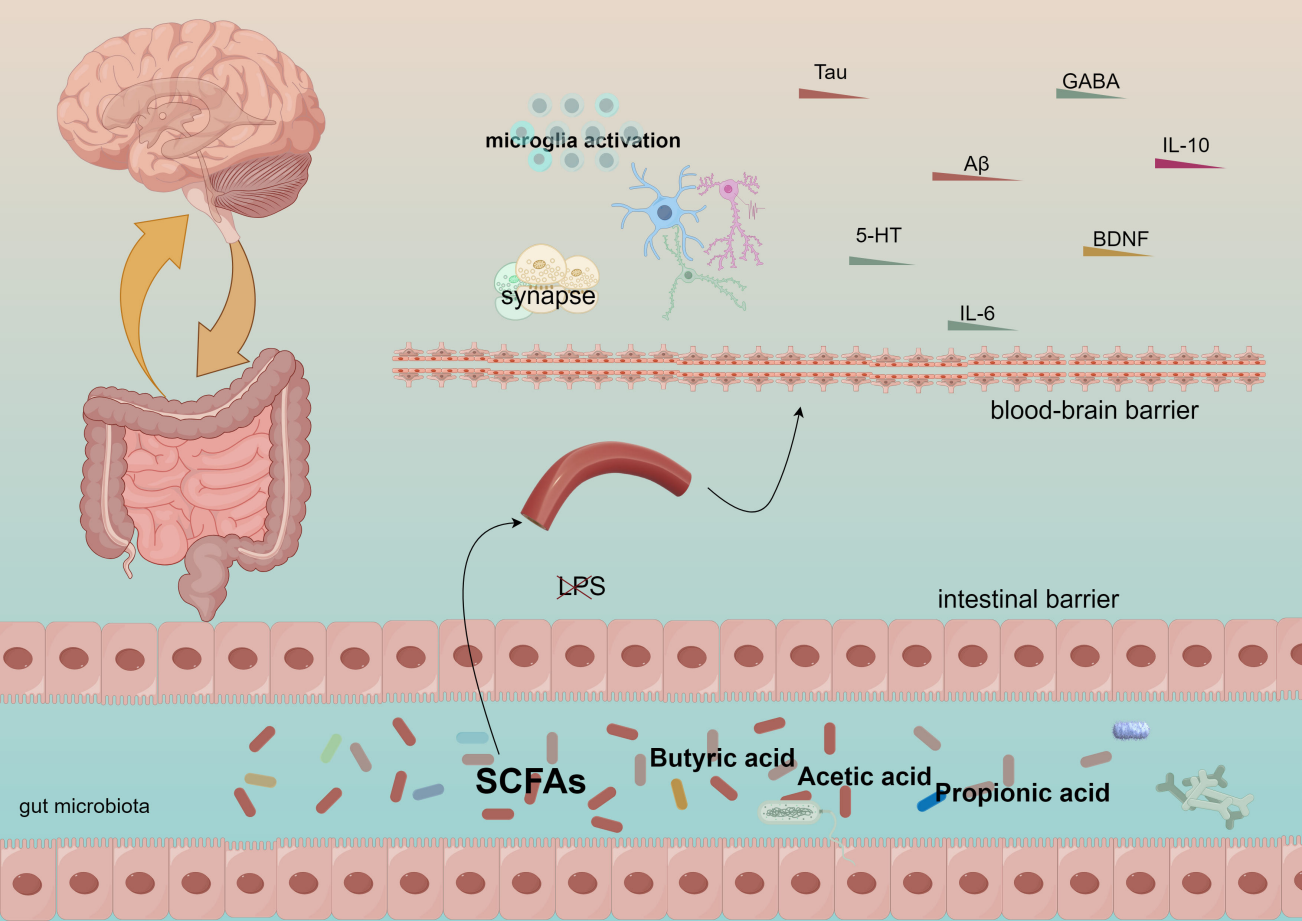
Short-chain fatty acids generate acetic acid, propionic acid, and butyric acid, which traverse the intestinal barrier and the blood-brain barrier, subsequently influencing the immune system and cognitive functions.

The neuroendocrine system plays a crucial role in the gut-brain axis (82). Gut-brain hormones, including leptin, may influence the perception of mental illness severity (83). Variations in leptin levels secreted by adipocytes can affect appetite regulation and energy metabolism, potentially leading to cognitive dysfunction in individuals with severe mental illnesses due to fluctuations in leptin levels (84). Furthermore, the neuroendocrine system regulates both the composition and function of gut flora, significantly impacting the gut-brain axis (83). For instance, under stress, the neuroendocrine system can modify the intestinal environment, resulting in an imbalance of gut flora that adversely affects cognitive function (84, 85). The gut microbiota can enter the bloodstream through short-chain fatty acids—such as acetic acid, propionic acid, and butyric acid—produced by the fermentation of dietary fiber, thereby influencing neural signaling in the brain (86). Notably, butyric acid, a metabolite of the gut microbiota, has been demonstrated to enhance neurogenesis and synaptic plasticity, positively impacting learning and memory processes (87). Short-chain fatty acids (SCFAs) can stimulate the secretion of mucus from intestinal epithelial cells through the modulation of the gut-brain axis. They enhance the integrity of the intestinal barrier, preventing the translocation of intestinal endotoxins, such as lipopolysaccharides, into the bloodstream. Furthermore, SCFAs influence the synthesis and metabolism of neurotransmitters and play a role in regulating neuroinflammation, as illustrated in **Figure 1**. They are crucial for maintaining the equilibrium of gut microbiota and impacting the metabolites produced by these microbial communities. Additionally, SCFAs promote neurogenesis and synaptic plasticity, facilitating the proliferation and differentiation of neural stem cells while improving synaptic transmission efficiency. Moreover, they exhibit antioxidant properties and support mitochondrial function, counteracting free radicals to safeguard the normal physiological activities of neurons and mitochondria. Additionally, the gut microbiota stimulates gut-associated lymphoid tissue (GALT), which influences the maturation and differentiation of immune cells, thereby regulating the systemic immune response (88). The composition of the gut microbiota is correlated with disease severity, and autoimmune diseases such as multiple sclerosis (MS) suggest that it may modulate the inflammatory state of the central nervous system (CNS) by affecting the immune response (89).

**Figure 2:**
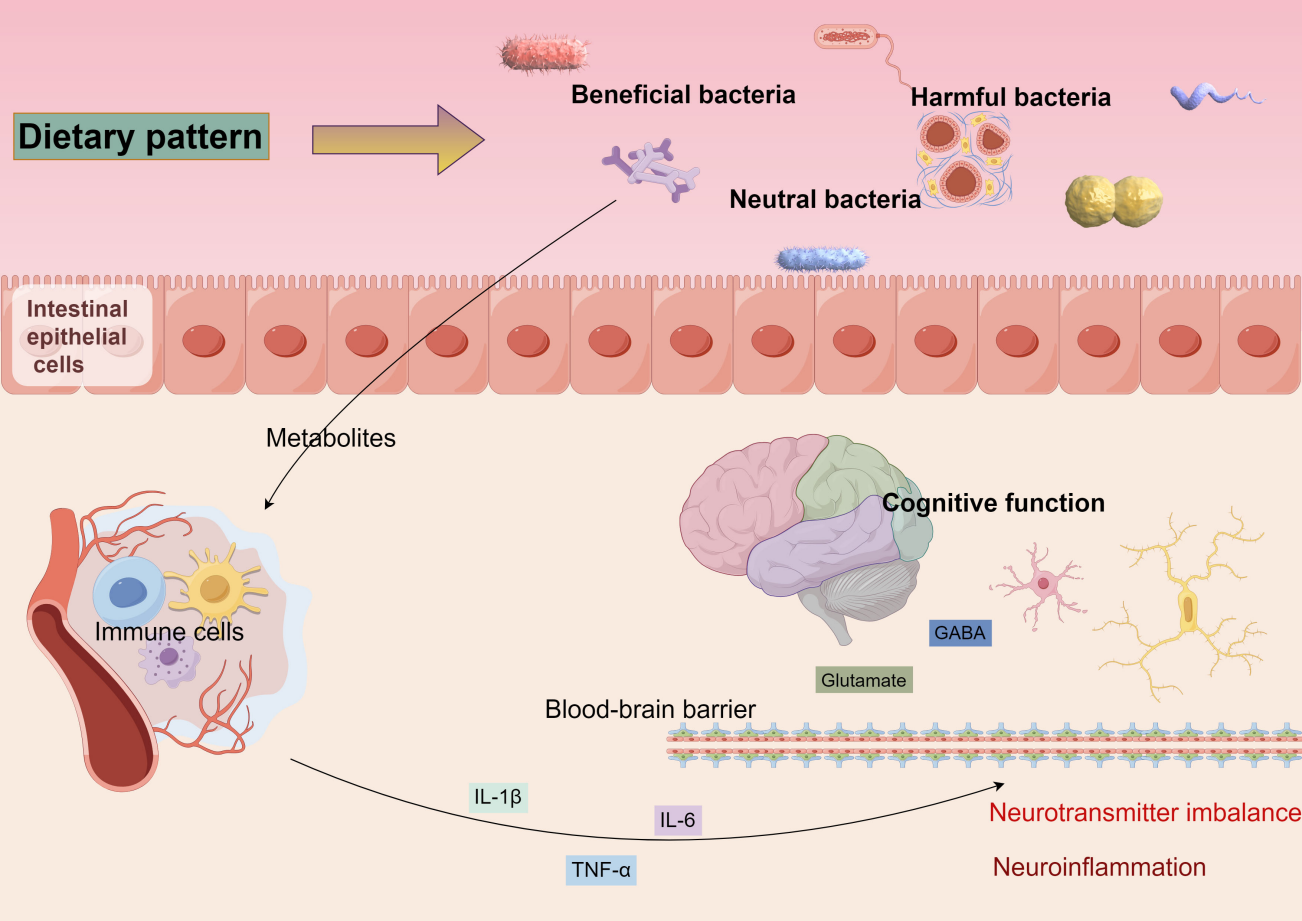
Diverse dietary patterns can alter the equilibrium of beneficial, detrimental, and neutral microbiota, leading to modifications in gut flora. These alterations can impact the integrity of the intestinal barrier, resulting in metabolites entering the bloodstream that activate the immune response. This activation prompts immune cells to secrete inflammatory mediators, which may traverse the blood-brain barrier, instigating neuroinflammation and disrupting neurotransmitter homeostasis, ultimately contributing to cognitive function alterations.

The mechanism of bidirectional gut-brain axis regulation refers to the brain’s role in regulating intestinal function while simultaneously influencing the composition and function of the gut microbiota (83). Stress and emotional states, mediated through the vagus nerve and hypothalamic-pituitary-adrenal (HPA) axes, impact the gut microbiota, which in turn affects cognition and mood (89, 90). Diet is another crucial factor shaping the composition of the gut microbiota; a high-fiber diet promotes the proliferation of beneficial bacteria and exerts anti-inflammatory effects, while a high-fat, high-sugar diet may disrupt this balance, triggering inflammation and autoimmune disorders that can impair cognitive function, as illustrated in **Figure 2**.

Research on the gut-brain axis offers new strategies for addressing cognitive dysfunction. Modulating the gut microbiota through probiotics, prebiotics, or fecal microbial transplantation (FMT) may enhance cognitive function (91). Furthermore, the development of drugs targeting specific gut microbial metabolites or immunomodulatory pathways represents a promising direction for future cognitive dysfunction treatments. In conclusion, the relationship between gut microbiota and cognitive dysfunction is a complex interplay involving multifactorial and multi-mechanism interactions. Future studies should further investigate the specific mechanisms by which gut microbiota influences cognitive function through the gut-brain axis and develop novel therapeutic strategies to enhance human health and cognitive performance.

## 5 The role of gut microbes in neurodegenerative diseases

Neurodegeneration is a multifactorial process influenced by genetic, environmental, and lifestyle factors, resulting in a progressive loss of neuronal structure and function (92). This deterioration gives rise to various neurodegenerative disorders, such as Alzheimer’s disease (AD), Parkinson’s disease (PD), and amyotrophic lateral sclerosis (ALS), which significantly debilitate individuals (93). The pathological features of these conditions encompass abnormal protein accumulation, mitochondrial dysfunction, oxidative stress, and neuroinflammation, ultimately leading to severely impaired cognitive and motor functions.

In recent years, researchers have recognized that the gut microbiota, a diverse community of microorganisms residing in the digestive tract, may influence the host’s nervous system through the gut-brain axis, potentially regulating neurodegenerative processes (94). The gut-brain axis encompasses complex interactions among the nervous, endocrine, and immune systems, with the gut microbiota communicating with the central nervous system through these pathways, thereby influencing the host’s behavioral and cognitive functions (74). Clinical studies have demonstrated that the abundance of the Mycobacterium anisopliae phylum in the gut of AD patients is decreased compared to that of the healthy population, while the abundance of the Mycobacterium thickum phylum is increased (95). Furthermore, the gut microbiota of patients with PD exhibits a reduced abundance of certain spore-producing bacteria, such as *Christensenaceae*, which is negatively correlated with the severity of motor dysfunction (96). Dysbiosis, characterized by disturbances in the composition and function of the gut microbiota, has now been associated with neurodegenerative pathologies (83), as illustrated in **Table 3**. Research has demonstrated that dysbiosis of the gut microbiota can result in neuroinflammation and compromise the integrity of the blood-brain barrier, disrupt the metabolism of neurotransmitters and neuromodulators, induce immune system dysfunction, increase oxidative stress levels, lead to the overproduction of reactive oxygen species (ROS), and impair neuronal and mitochondrial functions. For instance, the gut microbiota profiles of AD and PD patients differ significantly from those of healthy individuals, suggesting a possible causal relationship between microbial imbalance and disease progression (97). In AD patients, the abundance of inflammation-associated bacteria, such as *Enterobacteriaceae*, is elevated in the gut, whereas the abundance of bacteria with anti-inflammatory effects, such as *Bifidobacteria*, is diminished (98). Similar alterations have also been observed in patients with PD, indicating a potential relationship between changes in gut microbiota composition and the development of neurodegenerative diseases (99).

**Table 3:** The relationship between gut microbiota and cognitive dysfunction.

Name ofdisease	Changes in gut microbiota
Alzheimer's disease	Gut microbial diversity decreased significantly, with an increase in *Bacteroidetes*, a decrease in *Firmicutes*, a decrease in beneficial bacteria (such as *Lactobacillus* and *bifidobacterium*), and an increase in bacteria associated with inflammation (such as *Clostridium*)
Parkinson's disease	Gut microbiota composition differed from healthy controls, with a decrease in beneficial bacteria such as *Lactobacillus* and *Bifidobacterium*, an increase in bacteria associated with inflammation and neurodegeneration (such as *Proteobacteria*), and a significant decrease in *Prevotellaceae*
Autism spectrum disorder	The diversity of gut microbes has decreased, with a decrease in the genera *Prevotella* and *Faecalibacterium*, an increase in the genera *Clostridium* and *Desulfovibrio*, and an abnormal proportion of bacteria related to neurotransmitter metabolism
Mild cognitive impairment	Intestinal microbial diversity decreased, *Bacteroidetes* increased, *Firmicutes* decreased, short-chain fatty acid (SCFAs) producing bacteria decreased, and bacteria associated with inflammation increased
Multiple sclerosis	Intestinal microbial diversity decreased, with an increase in *Bacteroidetes*, a decrease in *Firmicutes*, a decrease in bacteria associated with immune regulation (such as *Ackermannia*), and an increase in bacteria associated with inflammation (such as *Proteobacteria*)
Depression	Gut microbial diversity decreased, bacteria associated with inflammation (such as *clostridium*) increased, beneficial bacteria such as *Lactobacillus* and *Bifidobacterium* decreased, and short-chain fatty acid (SCFAs) producing bacteria decreased
Anxiety disorder	The composition of the gut microbiome is abnormal, with a decrease in bacteria associated with neurotransmitter metabolism (such as *lactobacillus*) and an increase in bacteria associated with inflammation

Clinical data further confirm that gut barrier dysfunction is associated with disease progression in ALS patients (100). The disruption of gut barrier function can cause or exacerbate neuroinflammation by allowing microbial sub-metabolites to enter the brain via the circulatory system. Inflammation plays a crucial role in neurodegeneration (76). Pro-inflammatory cytokines, such as tumor necrosis factor beta (TNF-β), interleukin 1β (IL-1β), and interleukin 6 (IL-6), disrupt the blood-brain barrier (BBB), enabling peripheral immune cells to infiltrate the central nervous system and aggravate neuroinflammation (101). This inflammatory environment leads to neuronal damage and death, which is strongly associated with the pathological processes of neurodegenerative diseases, such as AD and PD. This is further substantiated by clinical data indicating early alterations in the microbiome of patients with Alzheimer’s disease (74). In addition, reduced estrogen levels following menopause can result in alterations to the gut microbiome’s composition, heightening the risk of systemic and neuroinflammation. Furthermore, an increased presence of pro-inflammatory gut microbiota in males may more profoundly trigger these neuroinflammatory mechanisms, thereby hastening neuronal impairment and cognitive deterioration (102).

**Figure 3:**
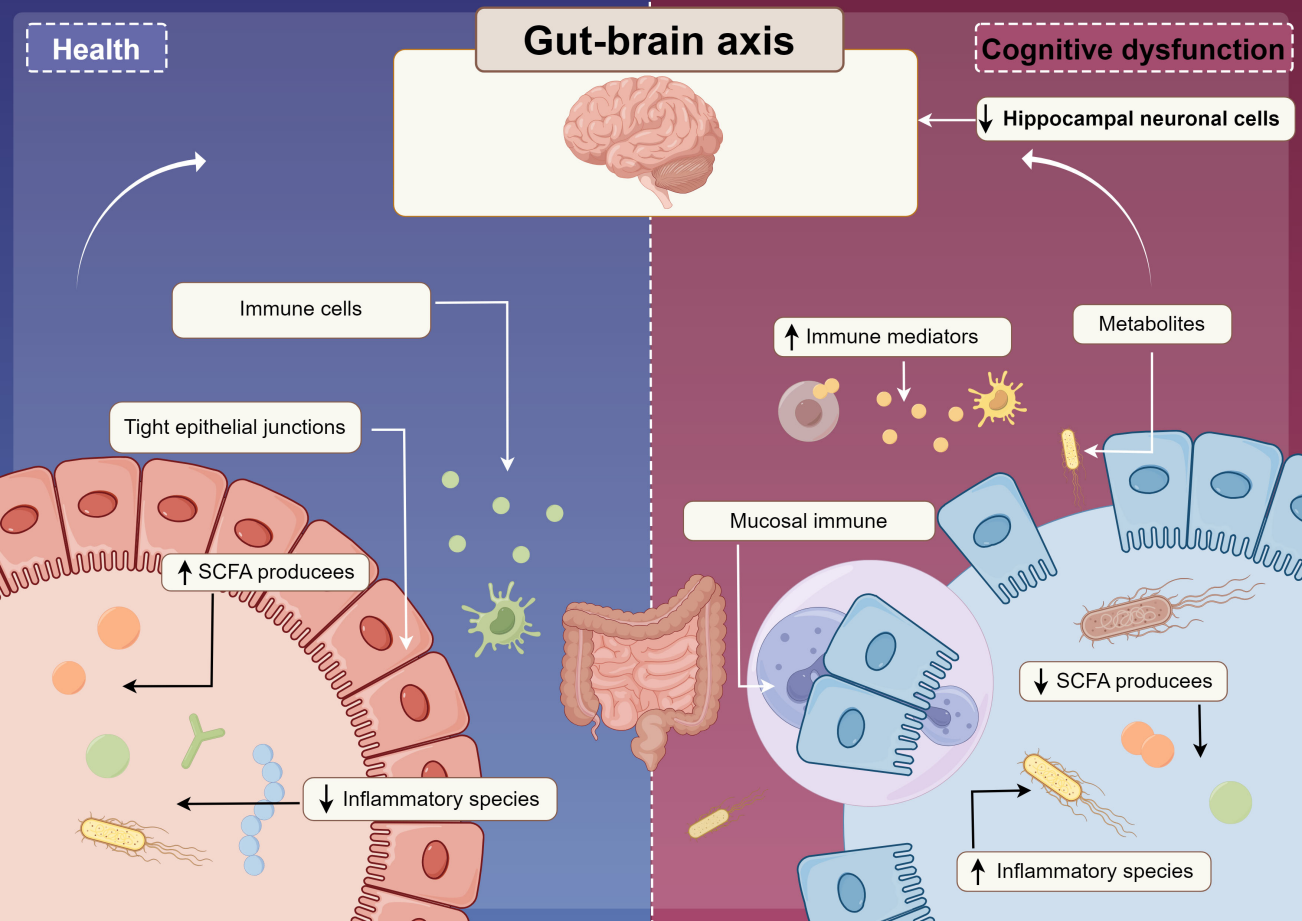
An intact gut, characterized by its tight junctions and well-regulated immune cell populations, is essential for the synthesis of short-chain fatty acids (SCFAs) and the mitigation of inflammation within the organism. Conversely, when the intestinal barrier is compromised, there is an elevation in immune mediators and metabolites, which subsequently activates an immune response in the intestinal mucosa and promotes inflammatory processes. These alterations, through their impact on hippocampal neurons, may ultimately contribute to cognitive impairments.

The anti-inflammatory effects of short-chain fatty acids (SCFAs) have been corroborated by clinical studies (103). For instance, a clinical trial demonstrated that cognitive function could be significantly improved by supplementing derivatives of butyric acid in patients with mild to moderate AD (87). Additionally, the production of neurotransmitters, such as gamma-aminobutyric acid (GABA), the primary inhibitory neurotransmitter in the central nervous system, and glutamate, the main excitatory neurotransmitter, is also influenced by the gut microbiota (81). By modulating the metabolic pathways of these neurotransmitters, the gut microbiota may significantly impact neurotransmission and synaptic plasticity, subsequently affecting cognitive and emotional functions. In summary, the gut microbiota may play a pivotal role in the development of neurodegenerative diseases by influencing the gut-brain axis through metabolite production and signaling, **Figure 3**. Future studies should further explore the mechanisms of interaction between the gut microbiota and neurodegenerative diseases and evaluate potential strategies to prevent or treat these conditions by modulating the gut microbiota.

## 6 Exploring the potential of using the gut microbiota as a therapeutic target

**Figure 4:**
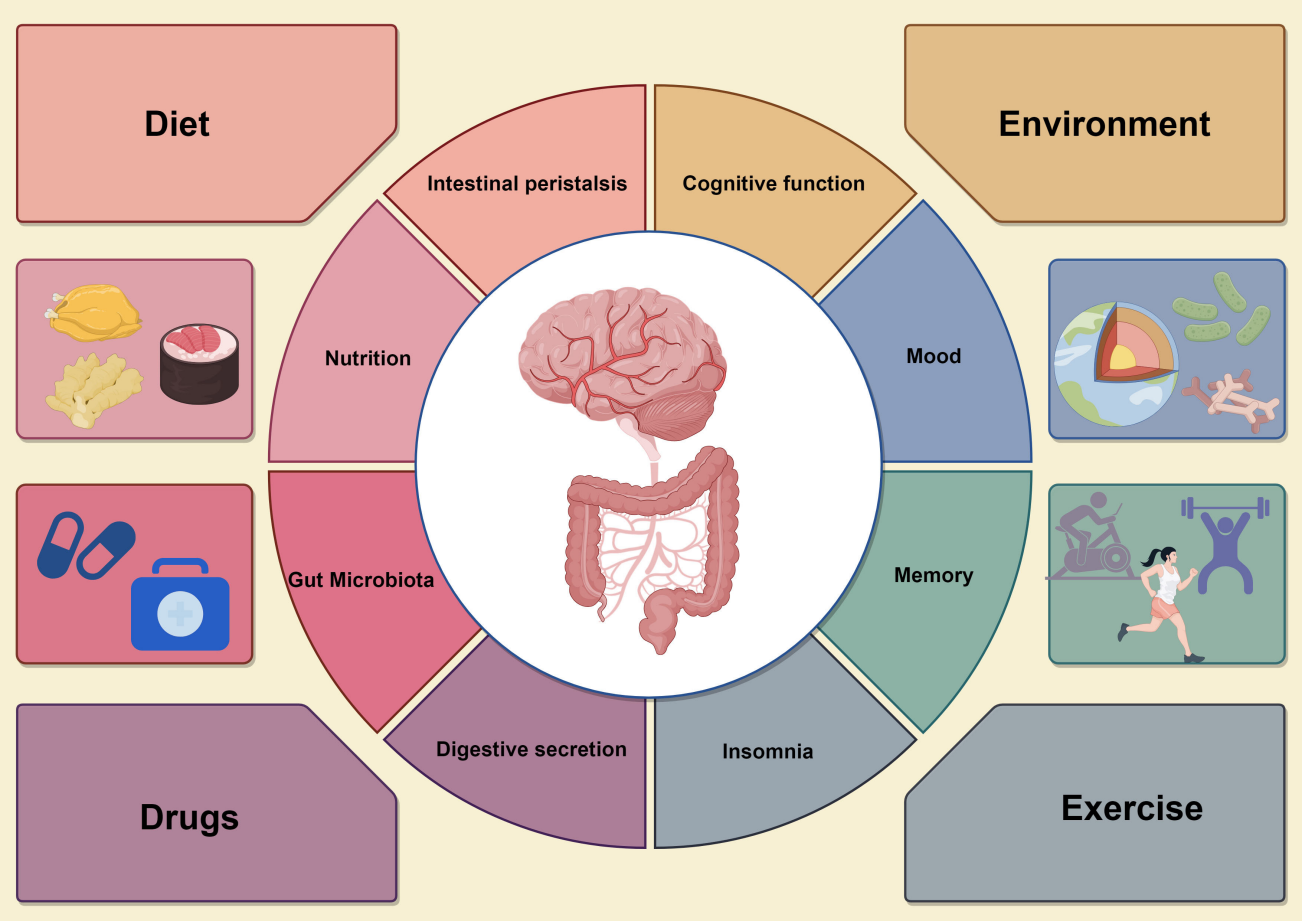
The circular diagram on the left shows the various effects of the gastrointestinal system, including microbiome balance, immune response, intestinal barrier integrity, and short-chain fatty acid (SCFA) production, all which support gut health. In contrast, the circular graph on the right illustrates how gut health affects neurological function, particularly cognitive processes, emotions, and neuroinflammatory responses. Dysbiosis in the gut microbiota can lead to increased pro-inflammatory mediators, impairing brain function and causing cognitive deficits. The four surrounding factors represent interventions to improve gut microbiota, such as dietary changes, probiotics and prebiotics supplementation, careful antibiotic use, and lifestyle changes like exercise and stress management. These strategies enhance both gut and brain health, promoting overall physical and mental well-being by optimizing gut microbiota composition and function.

The strong correlation between gut microbiota and human health is at the forefront of medical research, with these microbes playing crucial roles in digestion, immune system regulation, and cognitive function (104, 105). Consequently, they represent potential therapeutic targets for the treatment of immune-related diseases and cognitive dysfunction. In addressing these conditions, new strategies for adapting the composition and function of gut microbiota have been developed, **Figure 4**.

### 6.1 Dietary effects on gut microbiota

The impact of dietary patterns on gut microbes represents a comprehensive area of research that encompasses the function of the microbiome and its influence on host health through metabolites. Recent studies have demonstrated that gut microbial assemblages can undergo significant changes, which subsequently affect their function and metabolite production. For instance, different dietary habits, such as fiber-rich diets and high-fat diets, can lead to notable alterations in gut microbial composition (106). Fiber-rich diets have been shown to promote the production of short-chain fatty acids (SCFAs) by gut microbes, metabolites that play a crucial role in gut health and systemic immune regulation (66). Researchers have investigated the protein expression of gut microbes through controlled dietary interventions and have found that the composition and function of the microbiota can change rapidly in response to dietary modifications. These changes may have significant implications for drug metabolism, particularly concerning the capacity of certain microbial communities to metabolize drug components, thereby influencing drug bioavailability and efficacy.

Moreover, dietary patterns, by altering the metabolic pathways of gut microbes, may also be linked to various disease risks, including the association between gut microbes and colorectal cancer (CRC) risk (107). Fine-tuning gut microbes through dietary interventions may serve as a relevant tool for disease prevention and treatment. Thus, the impact of dietary patterns on gut microbes is a multifaceted process that affects gut health, overall immune response, and drug efficacy, influencing not only the type and abundance of gut microbes but also their metabolic activities. Future studies should further investigate the specific mechanisms by which dietary patterns affect the composition and function of gut microbes to provide more effective dietary guidance for the prevention and treatment of related diseases in clinical practice.

### 6.2 Influence of environmental factors on gut microbiology

In exploring the complex relationship between environmental factors and changes in gut flora, modern scientific research has revealed that various environmental factors—including diet, drugs, and environmental pollutants—affect the structure and function of the gut microbial community (108). Diet, being the most direct environmental factor, influences host health by altering the species composition of gut microbes and their metabolic activities, which subsequently impact host well-being. For instance, studies involving controlled diets have demonstrated that quantitative analysis of microbial protein expression reveals regulation of gut microbes by dietary patterns, evidenced by altered expression of microbial proteins, which in turn affects host metabolism (106). In addition to diet, regulating gut microbial composition is also significantly influenced by drugs. Research has shown that gut microbes not only influence the efficacy and toxicity of drugs through their metabolism but can also participate in the catabolism of drugs, thereby affecting drug metabolic pathways in the host. This interaction underscores the increasing importance of the gut microbial community as a critical consideration in individualized medical and therapeutic strategies.

Pollutants in the environment can adversely affect host health by altering the composition of gut flora (109). Recent studies have indicated that pollutants are associated with an increased risk of developing various diseases, including colorectal cancer, by disrupting the balance of the gut microbial community (108). Further research has demonstrated that gut microbiota, modified by specific environmental factors, can interact with host genetic factors to influence disease development. Notably, the establishment of gut flora during early infancy is significantly influenced by the mode of birth and early feeding practices, which have long-term implications for future health and development. Changes in the composition of gut flora at this stage can set the foundation for individual immune regulation and cognitive function (110).

In summary, environmental factors exert a multidimensional impact on the composition and function of gut microorganisms, leading to significant effects on host health. Future studies exploring the relationship between environmental factors, gut microbes, and host health, as well as the prevention and treatment of various diseases, are anticipated to yield new strategies.

### 6.3 Effects of drugs on gut microbiology

Drugs passing through the gastrointestinal tract may be altered by interactions with microorganisms, thereby affecting the efficacy and safety of the drug (111). A study by Malfatti et al. demonstrated that gut microorganisms can influence the biodistribution of drugs, particularly impacting the metabolism of acetaminophen (112). Furthermore, individual differences in microbial communities can lead to variations in drug response; Gurry et al. identified functional heterogeneity by examining the fermentation capacity of intestinal microbes in healthy populations, indicating that the composition of individual microbial populations may influence the impact of microbes on drug efficacy (113). The metabolic pathways of drugs within the organism are intricate, and their metabolism is characterized by complexity. The involvement of microorganisms can further diversify these metabolic pathways. Certain drugs can generate new metabolites that are subsequently metabolized by gut microbes. These microbial metabolites may exhibit varying biological activities, with some potentially enhancing the efficacy of the drug, while others may be toxic.

Gut microbes can establish a competitive relationship with drugs that influence drug absorption. As described by Madhusoodanan, microorganisms can compete for the intestinal absorption of the host by metabolizing drugs, which subsequently affects the blood concentration and efficacy of these medications (114). This phenomenon underscores the importance of microbiota in drug efficacy. The interactions between gut microbes and drugs significantly impact drug metabolism, absorption, efficacy, and even safety. Consequently, considering variations in the composition of individual gut microbiota during drug development may prove invaluable for personalized medicine. In the future, this approach could facilitate the creation of more precise drug treatment regimens that target the interaction mechanisms between specific drugs and gut microbes.

## 7 Conclusion

The gut microbiota is a crucial component of the intestinal microbiome and serves as a significant modulator of immune responses and cognitive functions. This review aims to synthesize the latest findings by examining the interactions between gut microbes and the host immune system, as well as their impact on cognitive impairment. The gut microbiota profoundly influences mucosal immunity and is essential for the development of immune tolerance and inflammation. Previous studies suggest the potential of microbiota-modulating therapies in shaping the immune response; however, the precise mechanisms by which microbial signaling translates into immune cell function require further investigation. Future research could provide therapeutic insights by elucidating the molecular pathways involved through genomics and proteomics techniques.

The role of short-chain fatty acids (SCFAs) in immunomodulation warrants further investigation, particularly regarding their interactions with other microbial metabolites, which remain unclear. SCFAs, such as butyric acid, are recognized for their contributions to anti-inflammatory effects. A deeper understanding of these metabolic interactions is crucial for the development of novel immunotherapies. The relationship between gut microbiota and cognitive disorders, often referred to as the gut-brain axis, is increasingly gaining attention. Metabolites like SCFAs are believed to influence brain function and behavior; however, the neural circuits and signaling molecules mediating these effects are not yet fully understood. Studies that integrate neuroimaging with microbiome analyses could provide valuable insights into how microbiological factors affect brain activity and cognitive performance. In the realm of neurodegenerative diseases, the notion of microbiota-induced inflammation as a contributing factor is receiving growing recognition. Nonetheless, the temporal relationship between microbial imbalances and neurodegenerative processes is complex and varies among individuals. Longitudinal studies that track changes in gut microbiota alongside neurological assessments may elucidate the temporal dynamics between microbial imbalances and neurodegenerative processes.

Gut microbiota therapy holds significant promise; however, challenges remain. The heterogeneity of individual responses to probiotics and other microbiota interventions suggests that a one-size-fits-all approach may not be effective. Personalized strategies that consider an individual’s unique microbiome profile, genetic background, and lifestyle factors are crucial for the success of these therapies. Furthermore, the influence of gut microbiota on drug metabolism introduces a new dimension to drug therapy, as variations in an individual’s response to the same medication may partially stem from differences in their gut microbiota. This necessitates a reassessment of how clinical trials and personalized treatment plans are designed. Additionally, environmental factors, including diet and exposure to pollutants, play a significant role in shaping the gut microbiota, and further investigation into how these factors can be manipulated to improve health outcomes is warranted.

Future research must investigate the molecular mechanisms by which the gut microbiota influences cognitive function through the gut-brain axis, focusing on specific microbial species, metabolites, and signaling pathways. Longitudinal studies are crucial to elucidate the causal relationship between alterations in the gut microbiota and cognitive function. Additionally, large-scale, randomized controlled clinical trials are necessary to enhance the efficacy and safety of interventions for cognitive dysfunction and to assess the modulation of the gut microbiota. Given the heterogeneity of gut microbiota among individuals, future studies should consider personalized therapeutic strategies tailored to individual gut microbiota profiles. Furthermore, it is vital to explore how the gut microbiota impacts drug metabolism and efficacy to inform personalized drug therapy. The development of new preventive and therapeutic strategies should also examine the influence of lifestyle factors—such as diet, exercise, and sleep—as well as environmental factors on the gut microbiota, and how these elements affect cognitive function through the gut-brain axis.

In summary, the role of the gut microbiota in immunomodulation and cognitive function represents a complex and dynamic field with significant potential for enhancing human health. This review explores the mechanisms through which the gut microbiota contributes to cognitive dysfunction via the immune system, while also addressing the limitations of the study and suggesting directions for future research. It is essential for upcoming research to integrate insights from diverse disciplines, including immunology, neurology, psychology, and nutrition, to fully leverage the potential of the gut microbiota in healthcare through a multidisciplinary approach. By deepening our understanding of the interactions between the gut microbiota and cognitive dysfunction, we anticipate the development of novel prevention and treatment strategies to tackle global public health challenges.
